# *ASPSCR1: TFE3* Fusion‐Positive Epithelioid Angiomyolipoma/PEComa Lacking Melanocytic Markers: A Case Report

**DOI:** 10.1002/iju5.70213

**Published:** 2026-06-21

**Authors:** Yuka Kondo, Yoji Nagashima, Kenji Zennami, Megumi Sato, Sayaka Sumiyoshi, Akihiko Sugimoto, Madoka Isomura, Kiyoshi Takahara, Sachiko Minamiguchi

**Affiliations:** ^1^ Department of Diagnostic Pathology, School of Medicine Fujita Health University Toyoake Aichi Japan; ^2^ Department of Surgical Pathology Tokyo Women's Medical University Hospital Tokyo Japan; ^3^ Department of Urology Nagoya University Graduate School of Medicine Nagoya Aichi Japan; ^4^ Department of Molecular Pathology, Graduate School of Medicine The University of Tokyo Tokyo Japan; ^5^ Department of Urology, School of Medicine Fujita Health University Nagoya Aichi Japan

**Keywords:** Angiomyolipoma, kidney neoplasms, PAX8 transcription factor, perivascular epithelioid cell neoplasms, TFE3 protein

## Abstract

**Introduction:**

Epithelioid angiomyolipoma is a rare perivascular epithelioid cell tumor subtype with malignant potential. Its morphology mimics renal cell carcinoma, posing diagnostic challenges. Half of these cases harbor *TSC1*/*TSC2* alterations, and a small subset features *TFE3* rearrangements, mutually exclusive of *TSC* mutations. Recently, “perivascular epithelioid cell tumor‐like neoplasms” with *ASPSCR1::TFE3* fusion lacking melanocytic markers were described.

**Case Presentation:**

A 25‐year‐old woman presented with a 4.5‐cm renal mass. Biopsy suggested *TFE3*‐rearranged renal cell carcinoma because of diffuse TFE3 positivity and no HMB45 immunoreactivity. Postoperatively, despite a complete lack of melanocytic markers, the tumor was diagnosed as *TFE3*‐rearranged epithelioid angiomyolipoma based on its epithelioid morphology and PAX8 negativity, including a retrospective biopsy study. Fluorescence in situ hybridization confirmed *ASPSCR1::TFE3* fusion, suggesting this rare, newly recognized entity.

**Conclusion:**

*TFE3*‐rearranged epithelioid angiomyolipoma can lack melanocytic markers. PAX8 immunohistochemistry and genetic testing help distinguish this potentially aggressive tumor from renal cell carcinoma and *TSC*‐mutated subtypes.

## Keynote Message

Distinguishing *TFE3*‐rearranged epithelioid angiomyolipoma from renal cell carcinoma is crucial. This case demonstrates that PAX8 negativity and genetic testing are essential for identifying melanocytic marker‐negative subtypes.

## 1 Introduction

Epithelioid angiomyolipoma (EAML) is a distinctive mesenchymal neoplasm belonging to the perivascular epithelioid cell tumor (PEComa) family. Although most are benign, EAML is recognized for its malignant potential [1]. EAML is a well‐known pitfall because its epithelioid morphology mimics renal cell carcinoma (RCC). The molecular pathogenesis of EAML commonly involves *TSC* *1* or *TSC* *2* expression. However, a small subset harbors *TFE3* gene rearrangements, which are mutually exclusive of *TSC* mutations [2]. *TFE3*‐rearranged tumors often occur in young patients without tuberous sclerosis complex (TSC) [3]. Differentiating *TFE3*‐rearranged EAML from *TFE3*‐rearranged RCC is challenging because both entities exhibit an epithelioid morphology and diffuse TFE3 immunoreactivity [4]. Recent studies have identified “PEComa‐like neoplasms” characterized by *ASPSCR1::TFE3* fusion without melanocytic marker positivity, further complicating the diagnostic landscape [5]. We report a case of *ASPSCR1::TFE3* fusion‐positive EAML that lacked melanocytic markers, which highlights the importance of PAX8 immunohistochemistry.

## 2 Case Presentation

A 25‐year‐old woman presented with incomplete bladder emptying. She had no medical or family history. Computed tomography (CT) revealed a 4.5 cm mass with an infiltrative margin, calcification, and marked contrast enhancement in the lower pole of the right kidney (Figure 1a,b), strongly suggesting RCC. However, given the patient's age and the atypical imaging features of the tumor, alternative diagnoses could not be excluded. Therefore, a CT‐guided biopsy was performed to establish a definitive diagnosis.

**FIGURE 1:**
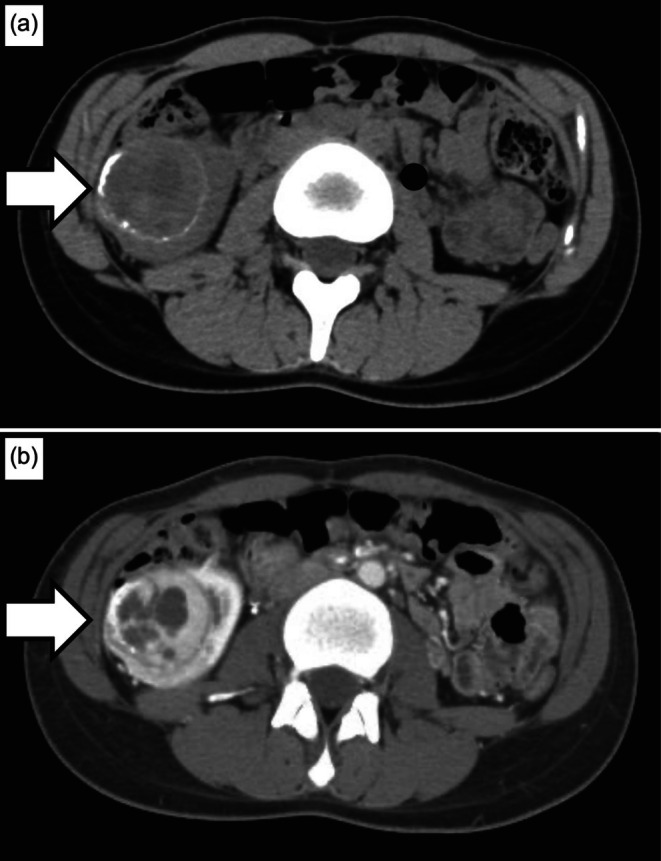
Computed tomography (CT) findings of the renal tumor. (a) Non‐contrast CT image showing a 4.5‐cm mass in the lower pole of the right kidney with an irregular outer margin accompanied by calcification (arrow). (b) Contrast‐enhanced CT (early phase) showing a hypervascular mass with early enhancement (arrow). The tumor also exhibited late‐phase washout (data not shown).

The tumor showed a solid growth pattern with nuclear pleomorphism and clear eosinophilic cytoplasm. The differential diagnoses included clear cell RCC, chromophobe RCC, and EAML. Immunohistochemistry (IHC) showed that the tumor cells were negative for CK (AE1/AE3), CK7, c‐kit, CAIX, and HMB45, diffusely positive for TFE3, leading to an initial diagnosis of *TFE3*‐rearranged RCC. Radical nephrectomy revealed a yellowish, solid tumor measuring 6.0 × 4.0 × 3.5 cm with hemorrhage, cystic change, and focal capsular invasion (Figure 2a,b). Histologically, the tumor was divided by fibrous septa and showed epithelioid growth patterns (Figure 2c). The tumor cells were round to polygonal with variable nuclear morphology and clear, eosinophilic, or vacuolated cytoplasm (Figure 2d,e). The results of IHC were the same as those of the initial biopsy specimen, and melan‐A was negative, thus maintaining the suspicion of *TFE3*‐rearranged RCC (Figure 3a). *TFE3* gene rearrangement was detected using break‐apart FISH (Figure 4a). However, additional IHC revealed that the tumor was negative for PAX8 and AMACR, but positive for cathepsin K with focal positivity for smooth muscle actin (Figure 3b,c). PAX8 negativity was also retrospectively confirmed in the initial biopsy specimen. Accordingly, the diagnosis was revised to *TFE3*‐rearranged EAML.

**FIGURE 2:**
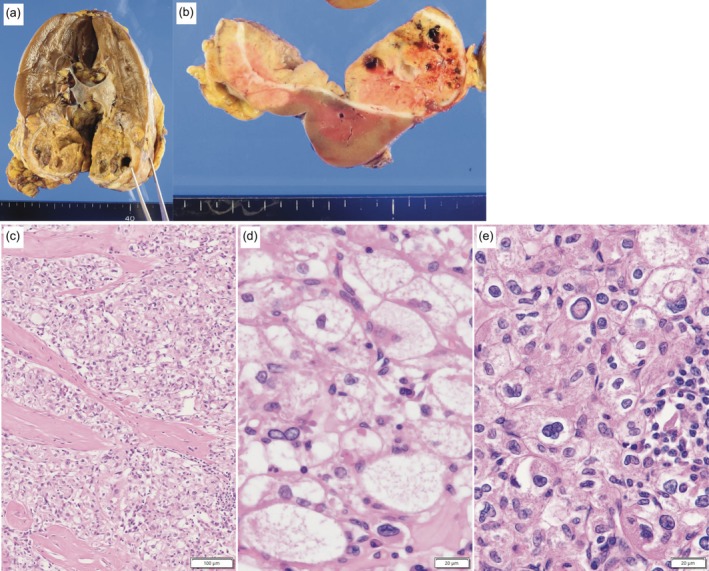
Macroscopic and microscopic findings. (a) The resected specimen showed a yellowish solid tumor. (b) The cut surface of the tumor exhibited hemorrhage, cystic changes, and focal capsular invasion. (c) Histologically, the tumor demonstrated a solid proliferation of epithelioid cells divided by delicate fibrous septa (H&E, ×200). (d,e) High‐power view revealed atypical polygonal cells with abundant clear to eosinophilic cytoplasm and prominent nucleoli (H&E, ×400).

**FIGURE 3:**
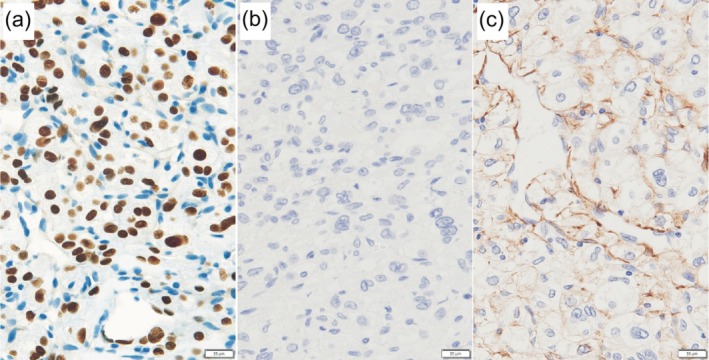
Immunohistochemical profile. The tumor cells exhibited (a) a diffuse and strong nuclear expression of TFE3 (×400). In contrast, the tumor cells were completely negative for (b) PAX8 (×400). The cells showed (c) focal positivity for smooth muscle Actin (×400).

**FIGURE 4:**
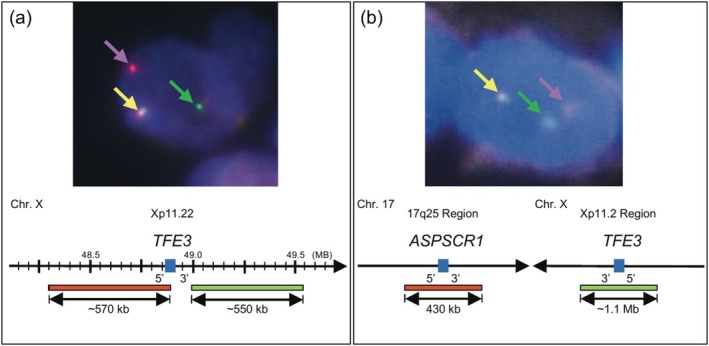
A molecular genetic analysis using fluorescence in situ hybridization (FISH). Bottom panels show schematic diagrams of the probe designs. (a) *TFE3* break‐apart FISH using a break‐apart probe flanking the *TFE3* gene (red: 5′ end, green: 3′ end). Separated red and green signals (arrows) indicate *TFE3* gene rearrangement. (b) Dual‐color fusion FISH for *ASPSCR1::TFE3*. Probes are designed to target the *ASPSCR1* locus (red) and the *TFE3* locus (green). An overlapping yellow signal confirmed the presence of the fusion gene.

Thereafter, a FISH analysis identified the *ASPSCR1::TFE3* fusion (Figure 4b). As the tumor completely lacked any melanocytic markers despite its EAML‐like morphology, this finding was consistent with the recently proposed category of “PEComa‐like neoplasm.” The postoperative course was uneventful for 16 months. No clinical signs of TSC were identified.

## 3 Discussion

*TFE3*‐rearranged EAML represents a rare subset of PEComas, typically occurring in younger patients and showing no association with TSC [3]. Unlike classic angiomyolipoma, EAML is characterized by the proliferation of atypical epithelioid cells and a malignant potential. The major clinical challenge is distinguishing *TFE3*‐rearranged EAML from *TFE3*‐rearranged RCC due to overlapping pathological and radiological features [4]. Radiologically, fat‐poor EAML mimics RCC [6]; even marginal (“eggshell”) calcification, often considered benign [7], occurs in *TFE3*‐rearranged neoplasms, such as Xp11.2 RCC and renal alveolar soft part sarcoma (ASPS) [8, 9]. Notably, *ASPSCR1::TFE3* tumors may exhibit stromal hyalinization or ossification [5], although any possible fusion‐specific correlations remain unclear.

To resolve these diagnostic challenges, PAX8 and cathepsin K are vital. PAX8 was negative in *TFE3*‐rearranged PEComas (0/88), but it was positive in 91% (216/237) of *TFE3*‐rearranged RCCs [4]. While cathepsin K is highly sensitive for *TFE3*‐rearranged PEComas (97%; 86/89), its specificity is limited by frequent positivity in *TFE3*‐rearranged RCCs (43%; 111/256), particularly those with *PRCC* or *SFPQ* fusions [10]. We therefore propose a diagnostic algorithm prioritizing PAX8 and cathepsin K as mandatory components; namely, PAX8 negativity reliably excludes RCC even in TFE3‐positive cases, melanocytic‐marker‐negative tumors where lineage determination is most challenging.

In the present case, the initial suspicion of RCC highlighted the limitations of TFE3 IHC as a stand‐alone diagnostic marker. A key finding against RCC was PAX8 negativity [10]. In addition, TFE3 IHC can yield false‐positive results depending on the fixation and staining conditions [11]. Therefore, confirmatory FISH is essential to verify the presence of a true *TFE3* rearrangement.

The complete absence of immunoreactivity for melanocytic markers further complicates the diagnosis. Argani et al. recently described a series of tumors termed “PEComa‐like neoplasms” harboring *ASPSCR1::TFE* *3* fusion, which is classically associated with ASPS [5, 12]. These neoplasms morphologically resemble EAML, but lack melanocytic differentiation. Our case is consistent with this emerging entity. The phenotypic variability associated with the same gene fusion likely reflects the “cell of origin” and the molecular strength of the fusion partner. PEComas may arise from neural crest precursors capable of melanocytic differentiation, while ASPS derives from mesenchymal stem cells lacking that potential [5]. Furthermore, *ASPSCR1::TFE3* might drive MiTF targets less effectively than the *SFPQ* partner [13]. However, a rare melan‐A‐positive *ASPSCR1::TFE3* case suggests a dynamic interplay between the cellular context and the fusion protein expression levels [14].

The pathological classification is clinically important because *TFE3*‐rearranged PEComas are thought to have a more aggressive clinical course than conventional EAML. Among the 42 reported cases with a follow‐up of more than 1 year, 13 (31%) developed systemic metastasis, and five (12%) died of the disease [12]. Recurrences and metastases typically occur within 2 years of the initial diagnosis, commonly involving the lungs, abdominal lymph nodes, and livers [4, 12]. Therefore, regular postoperative cross‐sectional imaging is essential in the follow‐up of such cases. The high‐risk features proposed by Argani et al. include a tumor size ≥ 5 cm, necrosis, and a mitotic count ≥ 1/10 high‐power fields. However, late‐onset metastasis has been reported as late as 7 years after resection, even in the absence of these features, thereby underscoring the need for long‐term oncological surveillance.

Thus, these molecular drivers have significant therapeutic implications. *TFE3* rearrangements and *TSC* *1/2* mutations were mutually exclusive [2]. Unlike *TSC*‐mutated PEComas, which may respond to mTOR inhibitors, *TFE3*‐rearranged PEComas appear to show limited sensitivity to these agents [12]. Therefore, identifying these tumors is essential for tailoring the postoperative management. Although Argani et al. proposed the term “*TFE3*‐rearranged PEComa‐like neoplasms” to emphasize their distinctive molecular background and limited sensitivity to mTOR inhibitors, this nomenclature has yet to be fully standardized in the current classifications. Given the rarity and phenotypic variability of “*TFE3*‐rearranged PEComa‐like neoplasms,” further accumulation of cases is therefore required.

## 4 Conclusion

*TFE3*‐rearranged EAML/PEComa may lack melanocytic markers. PAX8 negativity and FISH exclude *TFE3*‐rearranged RCC and conventional *TSC*‐mutated PEComa. It is essential to ensure appropriate long‐term surveillance to support the optimal selection of systemic therapy.

## Consent

We obtained consent from the patient for the publication of this case report.

## Conflicts of Interest

The authors declare no conflicts of interest.

## Data Availability

The data that support the findings of this study are available on request from the corresponding author. The data are not publicly available due to privacy or ethical restrictions.
